# Xanthan: enzymatic degradation and novel perspectives of applications

**DOI:** 10.1007/s00253-024-13016-6

**Published:** 2024-02-21

**Authors:** Oksana V. Berezina, Sergey V. Rykov, Wolfgang H. Schwarz, Wolfgang Liebl

**Affiliations:** 1 National Research Centre «Kurchatov Institute», Academician Kurchatov Sq. 1, 123182 Moscow, Russian Federation; 2 Chair of Microbiology, Technical University of Munich, TUM School of Life Sciences, Emil-Ramann-Str. 4, 85354 Freising, Germany

**Keywords:** Xanthan, *Xanthomonas*, Tailored biopolymers, Functional materials, Xanthan lyase, Xanthanase, *Bacillus*, *Microbacterium*, *Thermogutta*, Xanthan-utilizing gut consortia

## Abstract

**Abstract:**

The extracellular heteropolysaccharide xanthan, synthesized by bacteria of the genus *Xanthomonas*, is widely used as a thickening and stabilizing agent across the food, cosmetic, and pharmaceutical sectors. Expanding the scope of its application, current efforts target the use of xanthan to develop innovative functional materials and products, such as edible films, eco-friendly oil surfactants, and biocompatible composites for tissue engineering. Xanthan-derived oligosaccharides are useful as nutritional supplements and plant defense elicitors. Development and processing of such new functional materials and products often necessitate tuning of xanthan properties through targeted structural modification. This task can be effectively carried out with the help of xanthan-specific enzymes. However, the complex molecular structure and intricate conformational behavior of xanthan create problems with its enzymatic hydrolysis or modification. This review summarizes and analyzes data concerning xanthan-degrading enzymes originating from microorganisms and microbial consortia, with a particular focus on the dependence of enzymatic activity on the structure and conformation of xanthan. Through a comparative study of xanthan-degrading pathways found within various bacterial classes, different microbial enzyme systems for xanthan utilization have been identified. The characterization of these new enzymes opens new perspectives for modifying xanthan structure and developing innovative xanthan-based applications.

**Key points:**

*• The structure and conformation of xanthan affect enzymatic degradation.*

*• Microorganisms use diverse multienzyme systems for xanthan degradation.*

*• Xanthan-specific enzymes can be used to develop xanthan variants for novel applications.*

## Introduction

With the apparent negative consequences of the mass use of synthetic polymers, there is a growing need to replace them with natural materials. Carbohydrate-based biopolymers can complement or substitute synthetic materials in a variety of applications (Bilal and Iqbal 2019; Moradali and Rehm 2020; Berninger et al. 2021). The biodegradability of natural polymers is particularly advantageous in light of the escalating problem of accumulating non-degradable plastic waste and its environmental pollution.

**Fig. 1:**
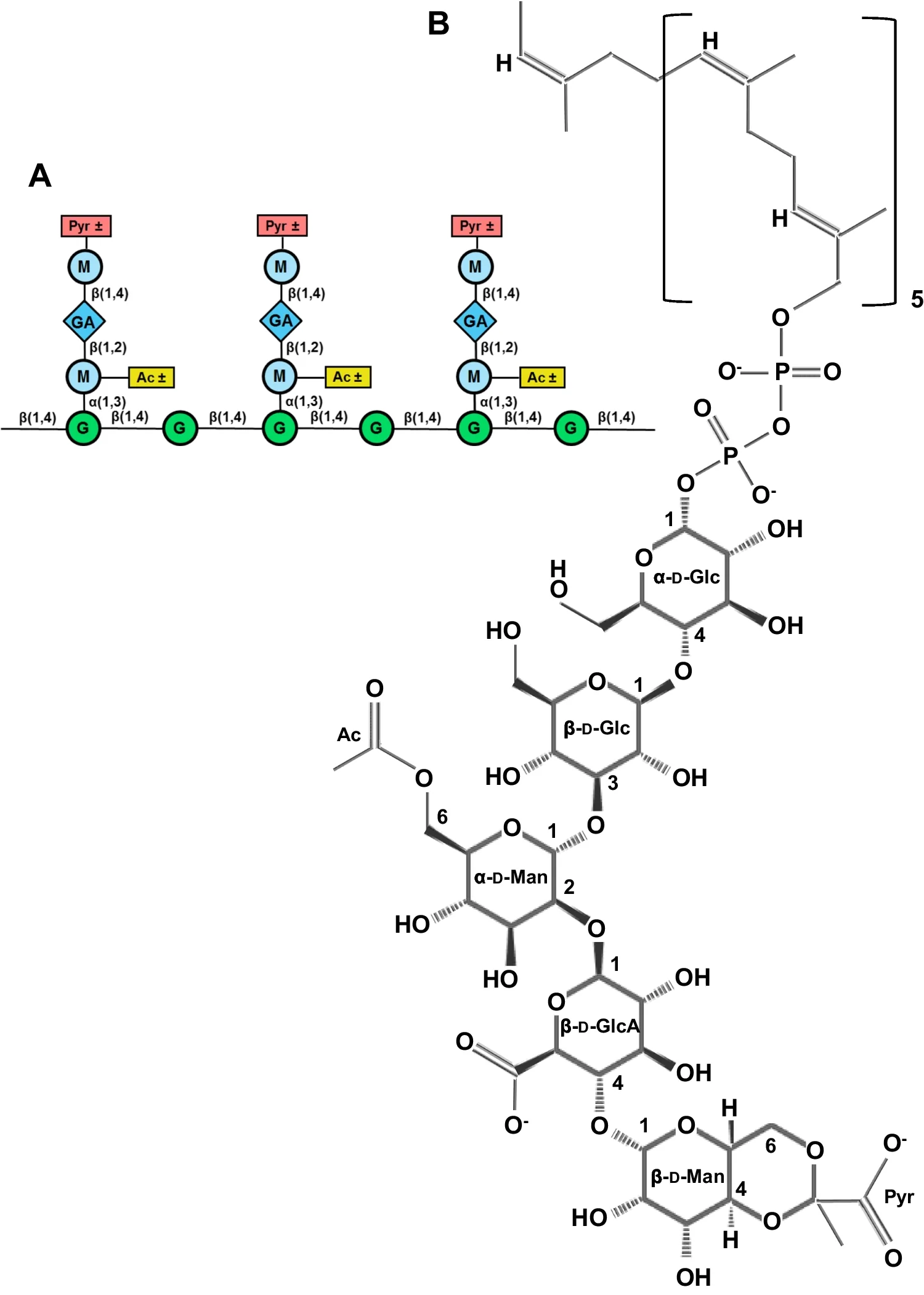
**A** Structure of a fragment of xanthan. G, glucose; GA, glucuronic acid; M, mannose; Pyr, pyruvoylation; Ac, acetylation; ± , variable. **B** Acetylated and pyruvoylated xanthan monomer 4,6-CH3(COO-)C-d-Man-β-(1,4)-d-GlcA-β-(1,2)-6-*O*-acetyl-d-Man-α-(1,3)-d-Glc-β-(1,4)-d-Glc-α-1-diphospho-ditrans,octacis-undecaprenol (PubChem CID 52940177)

Xanthan is an exopolysaccharide (EPS) synthesized by plant-pathogenic bacteria of the genus *Xanthomonas* (Petri 2015, Patel et al. 2020, Riaz et al. 2021). The xanthan capsule encloses the bacterial cell allowing it to adhere to the plant surface, to grow in a wide range of adverse conditions, and to avoid host defensive reactions (Bianco et al. 2016). The chemical structure of xanthan is complex due to the presence of various sugar constituents and associated chemical modifications (Fig. 1A) (Nsengiyumva and Alexandridis 2022).

This biopolymer has a number of remarkable properties. For instance, in salt-free aqueous solutions at room temperature, even at low concentrations (up to 1%), xanthan displays exceptionally high viscosity, reaching up to 10^6^ mPa·s, and exhibits pseudoplasticity. In addition, xanthan has stability across a broad range of temperatures, pH levels, and salt concentrations. It also can stabilize emulsions and form hydrogels. These characteristics, combined with its Generally Recognized As Safe (GRAS) status, have contributed to its extensive application across the food, pharmaceutical, household chemicals, and cosmetics industries (Petri 2015, Patel et al. 2020, Riaz et al. 2021, Nsengiyumva and Alexandridis 2022).

Ongoing research is primarily aimed at the development of novel xanthan-based products and functional materials for biomedical and technical applications (Petri 2015, Kumar et al. 2018, Patel et al. 2020, Riaz et al. 2021, Nsengiyumva and Alexandridis 2022). Furthermore, xanthan-derived oligosaccharides (XTOS), obtained by partial hydrolysis, have demonstrated their potential as food and feed supplements, antibiofilm agents, or elicitors of plant defense response (Xu et al. 2021; Wang et al. 2020; Qian et al. 2006, 2007). However, the development and manufacturing of such functional materials and products often require the alteration of xanthan’s molecular weight, solubility, or viscosity. In addition, depolymerization of xanthan is necessary for viscosity reduction in some technological processes, e.g., the petroleum industry, or for the environmentally safe disposal of industrial wastewater containing xanthan gum.

Xanthan can be modified or decomposed through enzymatic, physical, and chemical processes. Among them, the enzymatic approach is the preferred choice, primarily due to its specificity and minimal environmental impact, unlike alternative methods such as ultrasonic destruction, irradiation with ^60^Co γ-rays or hydrolysis using hydrogen peroxide in an alkaline solution (Patel et al. 2020, Riaz et al. 2021). However, the biodegradation of native xanthan gum is hindered by its double-stranded helical conformation, which exhibits varying degrees of structural order under different external conditions (Petri 2015; Riaz et al. 2021; Sutherland 1984; Kool 2014).

While elucidation of the precise mechanism underlying enzyme-xanthan interactions remains incomplete, significant progress has been made in recent years in studying the biology and enzymology of xanthan biodegradation. Surprisingly, despite its importance, a comprehensive review dedicated to xanthan biodegradation is still lacking in the literature. A recent review by Riaz et al. (2021) describes the main types of xanthan-degrading enzymes and provides a few illustrative examples, but the main attention of this study was on the comparison of enzymatic, physical, and chemical methods of xanthan modification, not on the microbial processes and enzymatic systems involved in the decomposition of xanthan. However, a thorough analysis encompassing both the fundamental and practical aspects of microbial degradation of xanthan is of value not only to enhance our understanding of this process, but also to promote the widespread utilization of xanthan-specific enzymes in various industries. A critical assessment of the current state of knowledge in this area is necessary to identify potential avenues for future research and practical applications.

Therefore, the primary objective of this review is to summarize and analyze the key findings concerning microorganisms utilizing xanthan, as well as the enzymes involved in its breakdown. Notably, recent discoveries of the xanthan-degrading pathways and related gene clusters in various classes of bacteria have led to significant progress in this area. Investigations into the enzymology of microbial xanthan degradation and comprehensive analysis of omics data uncovered a complex landscape, showing the absence of a uniform set of enzymes for xanthan decomposition but rather the existence of multiple degradation pathways in microbes.

The complexity and evolutionary diversity of mechanisms involved in the enzymatic degradation of xanthan possibly is a consequence of the complex structure of this biopolymer. Further studies of these mechanisms are necessary to optimize large-scale industrial processes of xanthan biodegradation and modification. Importantly, the diversity of degradative enzyme systems increases the number of enzyme candidates potentially useful for creating structural xanthan variants, which can be used for the development of innovative applications. This opens up new opportunities for using xanthan-degrading and modifying enzymes in diverse and promising ways.

## Innovative applications of xanthan and xanthan-based functional materials and products

### Applications of xanthan in the petroleum industry, agro-, and geotechnics

A number of xanthan’s characteristics have led to various applications unified by the superior viscosity characteristics of this polysaccharide. Xanthan synthesized by *Xanthomonas campestris* was approved for human consumption in the USA in 1969. It was the first microbial biopolymer to be produced commercially and has since become the largest-scale biopolymer produced. Apart from conventional applications as a stabilizer and thickener in various sectors of the economy, a substantial quantity of xanthan gum is utilized in the gas and petroleum industry. In most carbonate petroleum reservoirs, highly permeable zones are surrounded by an oil-wet matrix with low permeability. Extraction of oil from this rock matrix by waterflooding is significantly hindered (Gomari et al. 2021). Surfactants can modify the wettability of the matrix, thereby enhancing the recovery of residual oil. The rheological properties of xanthan make it a suitable ingredient for biodegradable surfactant blends employed in green enhanced oil recovery (GEOR) operations, for example, with an injection of a mixture of alkyl polyglucoside, xanthan, and butanone (Petri 2015, Tjon-Joe-Pin et al. 1997, Tjon-Joe-Pin and McClung IV 2018, Nsengiyumva and Aleksandridis 2022, Haq 2021).

Xanthan is a common component of drilling and well treatment fluids, including completion fluids, workover fluids, gravel packs, fracturing fluids, and blocking gels (Tjon-Joe-Pin et al. 1997; Tjon-Joe-Pin and McClung IV 2018). Xanthan solutions or mixtures thereof with other polymers are pumped into the wellbore during drilling to remove sludge from the well. For example, a blend of xanthan, the plant polysaccharide xyloglucan, and calcium phosphate as a weighting agent has demonstrated high pseudoplasticity and consistency, making it appropriate for use in water-based drilling fluids for horizontal wells (Petri 2015).

Due to its binding properties and gel-forming ability, xanthan is a promising additive for use in agro- and geotechnics. Studies have shown that xanthan can enhance water retention in soil, reduce evaporation, percolation, and soil erosion, and can replace synthetic polymers in drift control. Furthermore, xanthan has been found to protect encapsulated substances and regulate their release (Berninger et al. 2021). Cross-linked xanthan has been utilized to immobilize microorganisms for the biodegradation of naproxen in the environment (Dzionek et al. 2021). Xanthan also exhibits potential as an eco-friendly soil stabilizer and binder for road construction (Lee et al. 2019). Additionally, it can be added to self-curing concrete to enhance its viscosity (Tjon-Joe-Pin and McClung IV 2018).

### Applications of xanthan-based functional materials and composites

Xanthan-based functional materials and composites have broad prospects for use in various fields (Petri 2015, Patel et al. 2020, Kumar et al. 2018). In combination with other biopolymers, xanthan can be utilized to develop edible films and coatings that have advantages over synthetic plastics, such as biodegradability, safety, and production from renewable raw materials (Alizadeh-Sani et al. 2019). For example, a xanthan-curdlan composite may be superior in certain applications, e.g., as food packaging material, because of the excellent miscibility of its components, intermolecular bonding between the chains of xanthan and curdlan, the high tensile strength of the resultant films, and their enhanced solubility in water (Mohsin et al. 2020).

Xanthan-based hydrogels and cryogels are other types of functional materials with significant application potential. Cryogels are highly porous three-dimensional structures obtained by lyophilization of polymer solutions. They are effective in purifying water from pollutants. Hydrophobic cryogels made of silanized xanthan exhibit a high sorption capacity for mineral and sunflower oils and diesel. They also adsorb ethinyl estradiol from liquid media efficiently. Moreover, both hydrophilic and hydrophobic xanthan cryogels have been shown to efficiently remove bisphenol A, another estrogenic pollutant, from water. Two-layer “Janus” and multilayer monoliths, consisting of alternating hydrophilic and hydrophobic cryogels can be applied in mixtures of oil or diesel with water, which makes them highly versatile (Toledo and Petri 2019). An environmentally friendly anionic hydrogel composed of xanthan, cross-linked polyacrylic acid, and graphene oxide has also been developed for the purification of water from contamination with cationic dyes (Hosseini et al. 2020).

In medical applications, a flame-resistant hydrogel consisting of xanthan, starch, and resorcinol bis(diphenyl phosphate) (RDP) was shown to protect skin against burns. RDP is nontoxic to cells and is adsorbed directly onto polysaccharide delivery vehicles. Unlike water-based hydrogels, the xanthan/starch/RDP flame-retardation involves an endothermic reaction, making it highly effective (Xue et al. 2021). A thermo-reversible hydrogel consisting of xanthan and konjac glucomannan has been developed for wound dressing (Alves et al. 2020). A hydrogel prepared by thickening diphenyl diselenide solution with xanthan demonstrated an antitumoral effect against resistant melanoma cells. The formulation exhibited excellent permeation into the dermis layer and can be used as an adjuvant in melanoma medication (Ferreira et al. 2020).

In embryo biotechnology, a xanthan/locust bean gum gel can serve as an elastic culture substrate for in vitro embryonic environments. The development of bovine embryos cultivated on the gel was greatly improved (Hara et al. 2020).

Xanthan-based hydrogels and films also have attractive prospects for tissue engineering and regenerative medicine due to their mechanical properties, biocompatibility, and the possibility of implantation with minimal side effects (Kumar et al. 2018). For example, cytocompatible hydrogels formed by physical crosslinking of xanthan and silk fibroin possess enhanced water swelling capacity and suitable porosity and can imitate a cartilage extracellular matrix (Byram et al. 2020).

Biomimetic constructs made using hydrogels can accurately recreate the complex vascular architecture of living tissue (Correa et al. 2021). The combination of layer-by-layer assembly technology and bio-printing has enabled the design of complex scaffold structures with embedded cytocompatible microchannels mimicking blood vessels in vascularized tissues. The photocurable hydrogel composed of glycidyl methacrylated xanthan is a suitable material for the supporting matrix (Patrício et al. 2020; Sousa et al. 2021). The three-dimensional xanthan-based scaffold seeded with cells ensured the growth of vascularized tissue of controlled size and shape with improved mass transfer and cell viability (Sousa et al. 2021). This approach holds great potential for the bio-fabrication of complex tissue structures with enhanced functionality and physiological relevance.

The combination of chitosan and xanthan polysaccharides also can serve as a supporting matrix in soft tissue engineering. The addition of silicone rubber increased surface roughness and thickness without affecting the stability, biodegradation, thrombogenicity, and cytotoxicity of the supporting construct (Bombaldi de Souza et al. 2019). Composite films of xanthan and chitosan mineralized with hydroxyapatite are suitable for filling and repairing bone defects. Films impregnated with a solution of calcium hydrogen phosphate exhibited good results in vitro cell adhesion tests (Aguiar et al. 2019).

Functional materials based on xanthan can be used to create drug delivery systems (Cortes et al. 2020). Polysaccharide nanocontainers (NC) composed of a xanthan/diethylaminoethyl polyelectrolyte complex have been developed for the delivery of the lipophilic antitumor drug thymoquinone (Borodina et al. 2021). The complex does not show the lipophilic properties that often lead to high toxicity in antitumor drugs. Hydrophilic nanoparticles consisting of xanthan, sorbitan monooleate, and oleylamine were reported to bind to endothelial cells and to deliver incorporated plasmid DNA to the vascular endothelium of the kidneys, liver, and lungs (Fernandez-Piñeiro et al. 2018). These results highlight the potential of xanthan-based materials for the development of effective drug and gene delivery systems.

Xanthan-based graft polymers proved their potential for use in drug delivery, cell proliferation scaffolds, and environmental remediation (Maji and Maiti 2021). Biodegradable xanthan-grafted-poly(N-vinyl imidazole) has shown remarkable antibacterial properties against *E. coli* and *S. aureus*. Moreover, the grafted xanthan exhibited superior thermostability compared to the native xanthan (Elella et al. 2017).

### Applications of xanthan-derived oligosaccharides (XTOS)

XTOS have an unusually complex natural composition combining different sugars, chemical bonds, and modifications (Fig. 1). It is believed that this complex structure contributes to the observed biological activity of XTOS. It is noteworthy that XTOS have recently attracted considerable attention as plant defense elicitors. For example, XTOS produced using xanthan-degrading enzymes were shown to activate the synthesis of phytoalexin in soybean cotyledons and inhibit the growth of *X. campestris*. XTOS not only suppress the growth of the pathogen but also block the production of xanthan and extracellular enzymes such as proteinase, chitinase, cellulase, and pectinase by *X. campestris*. These findings suggest that XTOS may be an attractive alternative to unsafe chemical pesticides for protecting cruciferous plants from *X. campestris* (Liu et al. 2005; Qian et al. 2006, 2007).

In addition to their elicitor properties, XTOS have emerged as a promising supplement in the food and pharmaceutical industries due to their antibacterial and antioxidant properties. Low molecular weight XTOS (LM-XTOS) obtained by enzymatic degradation of xanthan have been shown to exhibit antibacterial activity against *Staphylococcus aureus* by increasing the permeability of the cell membrane and decreasing the level of transcription of genes responsible for biofilm development (Wang et al. 2020). Moreover, LM-XTOS have been found to mitigate oxidative damage caused by reactive oxygen species (Hu et al. 2019). XTOS with a degree of polymerization ranging from 2 to 15 sugar units have also demonstrated potential as prebiotics, promoting the growth of *Bacteroides* and butyrate-producing bacteria in vitro (Xu et al. 2021).

XTOS have also been investigated for their potential in the treatment of osteoarthritis. Injections of XTOS, similar to those of xanthan or the established medication sodium hyaluronate, were found in a rabbit model to protect articular cartilage from destruction and restore joint mobility. Furthermore, XTOS injections reduced oxidative stress-induced chondrocyte apoptosis and the concentration of NO in synovial fluid (Zhang et al. 2019). These results indicate that XTOS, like xanthan, is promising as a potential treatment for osteoarthritis.

## The relationship of structure, conformation, and viscosity of xanthan, and the influence of these factors on the susceptibility of the polymer to enzymatic attack

The success of various applications of xanthan depends on its resistance to biodegradation or, conversely, on effective decomposition (as discussed below). The chemical and physical structure of xanthan has a significant impact on its accessibility for enzymes, and therefore, understanding the enzyme-xanthan interactions is pivotal.

Referring to the comprehensive review of xanthan’s structure and its rheological behavior in aqueous solutions published by Nsengiyumva and Aleksandridis (2022), this section focuses in particular on xanthan’s structural properties concerning its susceptibility to enzymatic degradation. We aim to address several key questions: why is the viscosity of xanthan solution important for enzymatic decomposition, how does the viscosity depend on the conformation of xanthan, and what effect do external factors such as temperature and ionic strength have on the conformation of xanthan and thus on its accessibility to enzymatic attack.

The conformation of xanthan is determined primarily by its structure. The xanthan molecule has a β-(1,4)-d-glucan backbone, as in cellulose, with side chains of β-d-mannose-(1 → 4)-β-d-glucuronic acid-(1 → 2)-α-d-mannose which are connected to every second glucose residue by α-(1 → 3)-linkages (Holzwarth and Prestridge 1977) (Fig. 1A). During polymer biosynthesis in *Xanthomonas* cells, pentameric building blocks are assembled on an undecaprenyl-phosphate lipid carrier anchored in the cytoplasmic membrane (Becker 2015) (Fig. 1B). The terminal mannose in the side chains can be modified with (4,6)-linked pyruvate ketal (up to 65%) or acetyl groups (~ 15%), and about 90% of the inner mannose residues are *O*-6 acetylated (Kool 2014).

**Fig. 2:**
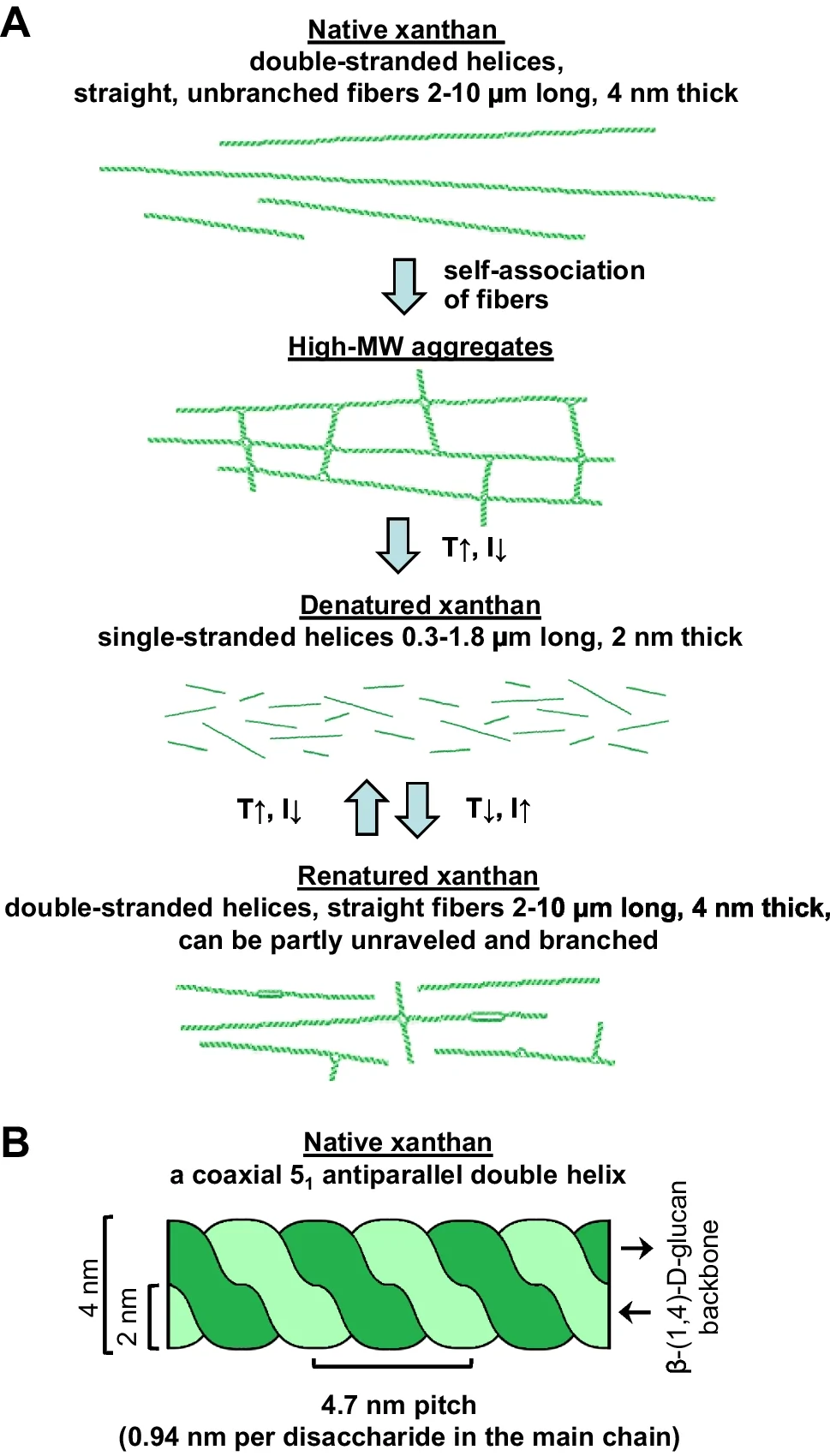
**A** Schematic representation of native, denatured, and renatured xanthan. **B** Xanthan in the native conformation exists in the form of coaxial 5_1_ antiparallel double helices (according to Holzwarth and Prestridge 1977)

Xanthan undergoes conformational alterations due to changes in environmental conditions such as ionic strength and temperature. The native xanthan, derived from the culture broth of *X. campestris*, consists of straight and unbranched fibers of 2–10-μm length and 4-nm thickness. These native xanthan fibers have a MW range of 4 to 20 × 10^6^, with an average MW of 15 × 10^6^ Da and a mass per unit length of approximately 1900 Da/nm. Upon denaturation, achieved through heating of dissolved native xanthan in a salt-free medium followed by rapid cooling, the fibers dissociate into shorter strands of typically 0.3- to 1.8-μm length and 2-nm thickness (Holzwarth and Prestridge 1977). An average denatured strand has a contour length of 0.5 μm, a MW of 0.5 × 10^6^, and a mass-to-unit-length ratio of 996 Da/nm, which is approximately half of its native counterpart (Fig. 2A). These observations suggest that a native double-stranded xanthan fiber, with a length of 10 μm, consists of roughly 40 smaller, single-stranded subunits (Holzwarth and Prestridge 1977; Holzwarth 1978). The existence of native xanthan fibers in the form of coaxial 5_1_ antiparallel double helices was confirmed by X-ray diffraction analysis (Holzwarth and Prestridge 1977; Morris 2017) (Fig. 2B).

The addition of 0.01–0.04 M NaCl induces the immediate renaturation of denatured xanthan into the double-stranded helical form, with single strands reassociating. It is noteworthy that renatured and native xanthan fibers have the same thickness. Although renatured xanthan is mostly unbranched, inter-chain associations can also lead to the formation of branched aggregates where neighboring xanthan chains connect (Holzwarth and Prestridge 1977; Kool 2014) (Fig. 2A).

According to the currently prevailing hypothesis, xanthan is synthesized as disordered coils, which then form single-stranded helices stabilized by their pyruvoylated side chains. Under suitable environmental conditions, these helices can further twist into double-stranded helices, which later self-associate to form polydisperse high-MW aggregates within the culture media (Southwick et al. 1980; Morris 2017). At high ionic strength, the charged trisaccharide side chains maintain a double-stranded helical conformation of xanthan. In salt-free aqueous solutions, the conformation of xanthan, and consequently its viscosity, distinctly depends on temperature. In the temperature range of 33 to 40 °C, the viscosity decreases, which is probably due to the destruction of the structure of high-MW aggregates. However, as the temperature continues to rise beyond this range up to 60 °C, the viscosity increases, indicating a disordering of the helical structure of native xanthan. At temperatures above 60 °C, the viscosity decreases again because under these conditions xanthan breaks up into short single-stranded disordered coils. Thus, thermal denaturation is the concurrent disruption of the aggregate structure and a change in the xanthan’s conformation. The complex rheological behavior of xanthan in aqueous solutions serves as a clear indicator of these alterations driven by changes in temperature (Southwick et al. 1980; Nsengiyumva and Alexandridis 2022).

In general, viscosity as a critical parameter reflects the conformational state of xanthan under specific environmental conditions, such as temperature and ionic strength. Moreover, viscosity affects the accessibility of xanthan to enzymatic degradation. Lowering the viscosity by thermal denaturation with the formation of single-stranded coils facilitates enzyme access to xanthan bonds. Conversely, the induction of a gel-like structure due to the formation of high-MW aggregates of double-stranded helices can protect xanthan against biodegradation. Finally, the viscosity of xanthan depends on its MW, and thus, the viscosity can potentially serve as an indicator of the extent of enzymatic decomposition of xanthan.

The conformation of xanthan can also be influenced by modifying its chemical structure. The composition of the side chains has a significant effect on the conformation and, consequently, on the viscosity of xanthan solutions. A modified xanthan derived from a mutant strain of *X. campestris*, with predominantly disaccharide side chains and a reduced level of pyruvoylation, demonstrated reduced viscosity. Treatment of this modified xanthan (polytetramer) with β-d-glucuronidase resulted in the formation of a polytrimer with a significantly higher viscosity. It is noteworthy that the acetylation of the polytetramer led to a decrease in its viscosity but had no effect on the polytrimer (Hassler and Doherty 1990).

The presence of acetyl groups on the side chains plays a crucial role in maintaining an ordered xanthan structure, whereas deacetylation leads to disordering, manifested in an increase in viscosity (Shatwell et al. 1990). Disordering promotes the formation of cross-linked composites between xanthan and other biopolymers. For example, the interaction between guar gum, a linear β-(1,4)-linked mannose chain with α-(1,6)-linked galactose residues, and deacetylated xanthan was stronger compared to native xanthan. Additionally, macromolecular association depends on the presence of pyruvoylation (Southwick et al. 1980; Khouryieh et al. 2007). Unlike native xanthan, the xanthan lyase-treated, depyruvoylated xanthan could not form a solid gel with locust bean gum, another galactomannan (Ruijssenaars et al. 1999, 2000) (Table 1).

**Table 1:** Enzymatic modification and degradation of xanthan

Modification of xanthan structure	Impact on the physical structure	Effects on characteristics	Enzyme(s) involved
Deacetylation of inner mannosyl residues	Disordering of the double-chain helix	Increasing the viscosity of the xanthan solution. Facilitating the formation of cross-linked composites between xanthan and other biopolymers	Acetyl esterase
Removal of pyrovoylated terminal mannosyl residues	Partial depolymerization (decomposition into subunits)	Partial decrease in viscosity of the xanthan solution. Macromolecular association and formation of cross-linked composites between xanthan and other biopolymers are difficult	Xanthan lyase
Removal of glucuronyl residues	Disordering of the double-chain helix	Increase in viscosity of the xanthan solution	Unsaturated glucuronyl hydrolase
Hydrolysis of xanthan backbone	Depolymerization to low-MW XTOS	Significant decrease in viscosity of the xanthan solution	Xanthan-specific endo-(1,4)-β-d-glucanase (xanthanase)
Hydrolysis of internal bonding in XTOS	Decomposition into sugar monomers	Complete saccharification of xanthan	β-Glucosidase, α-Mannosidase

Acetylation protects xanthan from degradation by depolymerizing enzymes, preventing the substrate-enzyme interaction. Deacetylation can be achieved enzymatically using esterases. However, due to the two different possible location sites of acetylation, the specificity of such enzymes and, consequently, their effect on xanthan viscosity may differ. For example, acetylxylan esterase AXE 3, obtained from the thermophilic fungus *Myceliophthora thermophila* C1, deacetylated exclusively the internal mannose residues in the side chains of xanthan, thereby stabilizing its disordered conformation (Kool 2014). In contrast, the acetyl pectin esterase YesY from *Bacillus subtilis* 168 deacetylated only the terminal mannose residues (Kool 2014). The effect of enzymatic deacetylation of mannose residues on the activity of depolymerizing enzymes remains an area requiring further study.

One might assume that disordering the state of xanthan would facilitate the access of depolymerizing enzymes to the β-(1,4)-glucan backbone. However, it is important to note that increased viscosity can potentially hamper the movement of the enzymes from one target site in the substrate to the next. Enzyme kinetics of the degradation of highly viscous polymeric substrates, such as xanthan, differ fundamentally from classical enzymology, which primarily deals with soluble substrates of low molecular weight that readily diffuse into the enzymes’ active site. The influence of viscosity on the kinetics of enzymatic decomposition of xanthan undoubtedly necessitates further investigation.

## Prospects for the application of xanthan-active enzymes

The relatively stable double-stranded conformation of native xanthan, along with its high viscosity, makes it highly resistant to enzymatic decomposition and potentially also to enzymatic modification. Nevertheless, the search for novel technologies utilizing xanthan and xanthan-derived products and materials requires the development of variants of this polymer with distinctive characteristics. The use of enzymes for this purpose can broaden the range of potential applications, for example, through the utilization of modified xanthan in composite materials with various other polymers (Hassler and Doherty 1990; Khouryieh et al. 2007).

As mentioned above, partial depolymerization by endo-acting microbial enzymes can be used for the production of functional XTOS. For instance, extracellular xanthan-degrading enzymes from the bacteria *Microbacterium* sp. XT11 and *Cellulomonas* sp. LX, or the fungus *Chaetomium globosum* have been employed to generate XTOS, exhibiting plant defense-eliciting and antibacterial activities (Liu et al. 2005; Qian et al. 2006, 2007; Wang et al. 2020). On the other hand, the use of decomposing enzymes is a valuable solution for the disposal of xanthan-containing waste obtained from various sources, including household chemicals, pharmaceuticals, and biodegradable xanthan-based materials such as films and coatings (Tjon-Joe-Pin and McClung IV 2018). Notably, xanthan-degrading enzymes have significant prospects in the oil and gas industry, given the widespread use of xanthan in surfactant blends, fluids for well treatment, and drilling fluids. Since the formation of xanthan-rich filter cakes and the leakage of xanthan-containing fluids decrease the permeability of the near-wellbore region, the injection of an enzyme solution can be used to reduce the viscosity of the fluid or to degrade polysaccharide cakes in wellbores or subsurface surroundings (Tjon-Joe-Pin et al. 1997; Tjon-Joe-Pin and McClung IV 2018).

Due to the high salt content in drilling fluids and the temperature in boreholes, often exceeding 60 °C, the use of thermostable enzymes has undeniable advantages. For example, xanthan-degrading enzymes produced by a consortium of the bacteria *Citrobacter freundii* and *Enterococcus faecalis* disintegrated xanthan filter-cake at temperatures above 65 °C with 85–96% efficiency. In comparison, the cleanup efficiency of conventional xanthan filter-cake breakers based on NaOCl and LiOCl treatment did not exceed 54% (Tjon-Joe-Pin et al. 1997; Tjon-Joe-Pin and McClung IV 2018). A soil strain, *Corynebacterium* 20/122, exhibited extracellular xanthan-degrading activity with a temperature maximum of 70 °C and therefore may be a promising source of thermostable enzymes for the oil industry (Cripps et. al 1979). Apart from wellbore applications, xanthan-degrading enzymes can also be employed in surface remediation of xanthan-containing drilling and well treatment fluids or degradation of xanthan-containing mud and deposits present in storage facilities such as containers, drums, drilling mud tanks, process vessels, etc. (Tjon-Joe-Pin et al. 1997; Tjon-Joe-Pin and McClung IV 2018).

Enzymes specifically acting on xanthan can also be used to modify structurally similar polysaccharides. For example, it has been shown that xanthan lyase releases glucuronic acid from the structure of EPS produced by bacteria of the genus *Rhizobium*, which leads to an increase in the thermal stability of the polysaccharide. *Rhizobium* EPS are used as stabilizers of fungal laccase or to counteract drought and prevent soil erosion. Improving the properties of the EPS after treatment with xanthan lyase expands the scope of possible applications of this polysaccharide (de Sousa et al. 2019).

The use of xanthan as a food additive in industrialized countries for more than 50 years has led to the adaptation of the human gut microbiota to the digestion of this polymer (Ostrowski et al. 2022). Therefore, the study of the microorganisms and enzyme systems that decompose xanthan in the human gut has important scientific and practical significance. Recombinant expression of xanthan-degrading and -modifying enzymes from this habitat could be an interesting resource for new applications.

## Biodegradation of xanthan

Despite the general resistance of xanthan to biodegradation, some organisms have developed the capability to decompose and utilize it. For example, numerous fungi can reduce xanthan viscosity using non-specific glycosidases. Fungal enzymes demonstrate optimal activity under environmental conditions favorable for the disordered conformation of xanthan, which emphasizes the importance of the xanthan structure for enzymatic access. On the contrary, some bacteria have evolved specialized multienzyme systems that allow stepwise degradation of xanthan, often starting with the decomposition of side chains. This destabilizes the ordered conformation of xanthan and allows enzymes to degrade its backbone.

### Degradation of xanthan by fungi

The capability to degrade xanthan was reported for the fungal genera *Aspergillus*, *Penicillium*, *Trichoderma*, and *Myceliophthora* (Sutherland 1984; Kool 2014; Tjon-Joe-Pin and McClung IV 2018). For example, enzyme mixtures from *Aspergillus niger* and *Trichoderma viride*, consisting of β-d-glucosidase, α- and β-d-mannosidase, and endo-β-glucanase, randomly hydrolyzed the xanthan molecule into d-glucose, oligosaccharides, and larger fragments (Sutherland 1984). The decomposition of the xanthan main chain was also obtained after treatment with a cellulase mixture from *M. thermophila* C1 (Kool 2014). However, a quantitatively noticeable hydrolytic degradation of xanthan by fungal cellulases was observed only in a salt-free solution, where the polymer is in a denatured conformation. Nevertheless, even after prolonged enzymatic treatment, polymer fragments resistant to hydrolysis with markedly increased acetate content were found (Cheetham and Mashimba 1991). This may be due to the clustered arrangement of acetylated residues in xanthan, contrasting with the random distribution of pyruvoylated or unsubstituted residues, which leads to an inhomogeneous ordering of xanthan fibers in aqueous solutions. (Kool 2014). The presence of the abovementioned enzymatic activities alone is thus not sufficient for successful degradation. Rather, specific and adapted enzymes are needed.

Deacetylation of the inner mannose residues and reduction of the content of side chains seems to increase the rate of xanthan hydrolysis by fungal cellulases, presumably due to the improved susceptibility of glucosidic bonds within the disordered xanthan. Depyruvoylation conceivably disrupts the double-stranded configuration of native xanthan. This can also improve the approach of cellulases to the polymer chain, thereby increasing the rate of hydrolysis, which, however, remains lower than for deacetylated xanthan (Cheetham and Mashimba 1991).

Thus, the rate of xanthan hydrolysis by fungal enzymes depends on the level of local polymer ordering and is predominantly constrained by the double-stranded helix conformation. Disruption of this conformation significantly promotes the depolymerization of xanthan. In this respect, xanthan exhibits similarities to cellulose, where disordered fragments are more susceptible to hydrolysis compared to the ordered crystalline regions (Leis et al. 2018).

### Degradation of xanthan by bacteria

Xanthan-utilizing bacteria are the richest reservoir of enzymes capable of degrading and modifying xanthan. Most of these bacteria known to date belong to the class *Bacilli* and are found in the genera *Bacillus*, *Anoxybacillus*, *Paenibacillus*, *Brevibacillus*, *Cohnella*, and *Enterococcus.* Along with *Bacillaceae*, the family *Paenibacillaceae* is a rich source of xanthan-utilizing strains. In addition, strains capable of decomposing xanthan were found in the genera *Cellulomonas*, *Microbacterium*, *Corynebacterium*, *Verrucomicrobium*, *Enterobacter*, *Citrobacter*, and the families *Thermoguttaceae* and *Oscillospiraceae* (syn. *Ruminococcaceae*), (Table 2).

**Table 2:** Xanthan-degrading microbial strains and the mixed cultures obtained by enrichment on media containing xanthan as a sole carbon source

Class	Order	Family	Genus	Strain (isolation source)	Xanthan-degrading activity	Xanthan degradation products	References
*Bacilli*	*Bacillales*	*Bacillaceae*	*Bacillus*	*Bacillus* sp. SB II (soil, Canada)	Extracellular xanthan-depolymerase and xanthan lyase activities. T_opt_ 40 °C, pH_opt_ 6.0–7.0	Low-molecular-weight XTOS with Δ4,5GlcA moieties	Lesley 1961
				*Bacillus* sp. K11 (NRRL B-4529), (soil inside a decaying tree trunk, Iowa City, USA)	Extracellular salt-tolerant xanthan-degrading enzymes produced by the B-4529 and B-4530 were identical. B-4530 yielded twice the amount of enzymes produced by B-4529. T_opt_ 48 °C in absence of NaCl and 42 °C in presence of 0.1 M NaCl, pH_opt_ 5.4. 100% activity retained after 30 min at 38 °C or in presence of 0.25% xanthan at 42 °C. 90% activity retained after 30 min at 42 °C	Uronic acid, mannose, pyruvoylated mannose, acetylated mannose, and β-(1,4)-D-glucan. Reduction of xanthan viscosity, increasing the reducing sugar level	Cadmus et al. 1982
				A mixed culture NRRL B-4530 consisted of *Bacillus* sp. K11 and *Flavobacterium sp*. K17 (NRRL B-14010), (soil inside a decaying tree trunk, Iowa City, USA)			
				*Bacillus* sp. 13–4 (NRRL B-4533), (sewage sludge, Peoria, USA)	Extracellular salt-sensitive xanthan-degrading activity. T_opt_ 42 °C, pH_opt_ 5.4. 100% activity retained after 30 min at 42 °C		
				A mixed culture HD1 of salt-tolerant aerobic microorganisms with a *Bacillus* sp. strain (soil, Linden and Summit, USA)	Extracellular xanthan-degrading activity. T_opt_ 35–45 °C; T_inact_ 50 °C. Xanthan viscosity reduction at pH_opt_ 5.0; reducing sugars formation at pH_opt_ 6.0	Glucose, glucuronic acid, mannose, pyruvoylated mannose, acetylated mannose, oligo- and polysaccharides	Hou et al. 1986
				*Bacillus* sp. strain GL1 (closely related to *Brevibacillus thermoruber*) (soil of paddy field, Japan)	Extracellular xanthan lyase and β-d-glucanase; intracellular α-d-mannosidase, unsaturated glucuronyl hydrolase, and β-d-glucosidase	Pyruvoylated mannose, Δ4,5GlcA, acetylated mannose, glucose	Hashimoto et al. 1998, Nankai et al. 1999
				*Bacillus* sp. strain R (water of local streams and ponds, Edinburgh, UK)	β-Glucosidase, α-mannosidase, xanthan lyase, xanthan- and CMC-specific β-glucanase activities	Pyruvoylated mannose, glucose, linear XTOS containing terminal mannose or Δ4,5GlcA	Sutherland 1982, 1987
				*Bacillus pumilus* (high-salt recovery water in the oil industry, Gulf of San Jorge, Peru)	Extracellular xanthan-degrading activity	Reduction of xanthan viscosity;	Gimenez et al. 2018
			*Anoxybacillus*	*Anoxybacillus flavithermus* and *Anoxybacillus rupiensis* (DSM 17127 T, NBIMCC 8387 T)—aerobic thermophiles (up to 60 °C), (soil, water, and algobacterial mat of hot springs, Bulgaria, Rodopa mountain)	Thermostable enzymes involved in xanthan degradation and utilization	Identification of products was not carried out	Derekova et al. 2007, 2008
		*Paenibacillaceae*	*Brevibacillus*	*Brevibacillus thermoruber—*aerobic thermophilic (up to 60 °C), (soil, water, and algobacterial mat of hot springs, Bulgaria, Rodopa mountain)	Thermostable enzymes involved in xanthan degradation and utilization	Identification of products was not carried out	
			*Cohnella*	*Cohnella.* sp. 56 (VKM B-3672D), (xanthan solution at a food production facility, St. Petersburg, Russia)	Extracellular xanthan-degrading activity at 50 °C, pH 7.2	Reduction of viscosity of xanthan-containing media during the strain cultivation	Denisenko et al. 2022
			*Paenibacillus*	*Paenibacillus taichungensis* I5 (VKM B-3510D), (xanthan solution at a food production facility, St. Petersburg, Russia)	Extracellular xanthan-degrading activity at 50 °C, pH 7.2	Reduction of viscosity of xanthan-containing media during the strain cultivation	Denisenko et al. 2020
				*Paenibacillus alginolyticus* XL-1 (soil, Netherlands)	Extracellular uronic acid-releasing activity and xanthan lyase activity on unmodified or deacetylated xanthan induced by xanthan, inhibited by glucose and XTOS. T_opt_ 55 °C, pH_opt_ 6.0. Endo-glucanase, β-mannosidase, α-mannosidase, β-glucuronidase, and β-glucosidase activities were not detected	Intact xanthan backbone, pyruvoylated mannose, uronic acids, uronic acid-containing oligosaccharides	Ruijssenaars et al. 1999
				*Paenibacillus nanensis* AS 5, *Paenibacillus agaridevorans* AS 8, *Paenibacillus phyllosphaerae* AS 9, *Paenibacillus agarexedens* AS 10, and *Paenibacillus taohuashanense* AS 26 (soil and sun-dried benthic sludge (Urmia Lake, Iran)	Extracellular xanthan-depolymerase and xanthan lyase activity with different levels among the isolates. T_opt_ 35–45 °C; pH_opt_ 5.0–7.0. All strains showed maximum activity in absence of NaCl, except for AS5 showing maximum activity up to 0.1 mol/L NaCl at 45 °C; 40% activity of AS5 retained at 50 °C and 60% at pH 4.0	XTOS and simple sugars	Ashraf et al. 2017
				*Paenibacillus xanthanilyticus*, isolate AS7 (IBRC M 10987 T, LMG 29451 T) (soil and sun-dried benthic sludge, Urmia Lake, Iran)	Extracellular xanthan-depolymerase and xanthan lyase activity. T_opt_ 40 °C. 60% and 20% of activity retained at pH 10 and 11 respectively	XTOS and simple sugars	Ashraf et al. 2018
				*Paenibacillus* sp. 62,047 (99% 16SrDNA identity to *P. nanensis* DSM 22867) (forest soil, China)	Extracellular xanthan-degrading activity	Identification of products was not carried out	Moroz et al. 2018
				*Paenibacillus* sp. XD CCM 7580*,* (activated sludge of a local municipal wastewater treatment plant, Czech Republic)	Xanthan and alginate utilizing enzymes	Reduction of xanthan viscosity; identification of products was not carried out	Muchová et. al. 2009
	*Lactobacillales*	*Enterococcaceae*	*Enterococcus*	*Enterococcus faecalis* (soil consortium ATCC 55941)	Extracellular thermostable xanthan-degrading enzymes	XTOS	Tjon-Joe-Pin et al. 1997
*Gammaproteobacteria*	*Enterobacterales*	*Enterobacteriaceae*	*Citrobacter*	*Citrobacter freundii* (soil consortium ATCC 55941)	Extracellular thermostable xanthan-degrading enzymes	XTOS	
			*Enterobacter*	*Enterobacter* sp. nov. LB37, salt tolerant (soil, Dalian, China)	Extracellular xanthan-degrading activity induced by cell growth on xanthan, maltose, soluble starch, sucrose, lactose, CMC, ethanol, mannose, and mannitol but except for agar and alginate	Reduction of xanthan viscosity; identification of products was not carried out	Chen et al. 2013
*Verrucomicrobiae*	*Verrucomicrobiales*	*Verrucomicrobiaceae*	*Verrucomicrobium*	*Verrucomicrobium* sp. CCM 7579 (isolated by enrichment on gellan from activated sludge of a local municipal wastewater treatment plant, Czech Republic)	Xanthan and gellan-utilizing enzymes	Reduction of xanthan viscosity; identification of products was not carried out	Muchová et. al. 2009
*Actinomycetia*	*Micrococcales*	*Cellulomonadaceae*	*Cellulomonas*	*Cellulomonas* sp. LX (soil, Dalian, China)	Extracellular xanthan-degrading activity induced by xanthan and inhibited by glucose in a concentration of more than 600 mg/L. T_opt_ 40 °C, pH_opt_ 6.0	XTOS	Liu et al. 2005, Qian et al. 2006
		*Microbacteriaceae*	*Microbacterium*	*Microbacterium* sp. XT11 (related to *Microbacterium hydrocarbonoxydans* and *Microbacterium paraoxydans*), salt tolerant, (soil of the plant garden, Dalian, China)	Extracellular and intracellular enzymes involved in xanthan degradation and utilization induced by xanthan or to a lesser extent, CMC and partially inhibited by glucose	XTOS	Qian et al. 2007, Yang et al. 2016, Sun et al. 2019
	*Mycobacteriales*	*Corynebacteriaceae*	*Corynebacterium*	*Corynebacterium* sp. 20/122 (NCIB-11535) (soil, Netherlands)	Extracellular xanthan- and CMC-degrading activity at 25–70 °C and a pH 5–7. T_max_ 70 °C, pH_max_ 5.6. After 1-h period, 30% of activity was lost at 30 °C, 50% lost at 45 °C, and 100% at 50 °C	Depolymerisation of deacetylated xanthan into mannose; pyruvoylated mannose; linear tetrasaccharide containing Δ4,5GlcA; pentasaccharide	Cripps et. al 1979
*Clostridia*	*Eubacteriales*	*Ruminococcaceae*	Not determined	An uncultivable bacterium from the genus 13 of the family *Ruminococcaceae* (R.UCG13) (human gut microbiota)	Extracellular and intracellular enzymes involved in xanthan degradation and utilization	XTOS produced by R.UCG13 were further degraded by *B. intestinalis*	Ostrowski et al. 2022
*Bacteroidia*	*Bacteroidales*	*Bacteroidaceae*	*Bacteroides*	*Bacteroides intestinalis* identified in human gut microbiota	Extracellular and intracellular enzymes involved in XTOS degradation and utilization		
*Planctomycetia*	*Pirellulales*	*Thermoguttaceae*	*Thermogutta*	A thermophilic anaerobe *Thermogutta terrifontis* R1 (60 °C) (microbial mat of a terrestrial hot spring of Kunashir island, Far-East of Russia)	Thermostable extracellular and intracellular enzymes involved in xanthan degradation and utilization	Experimental identification of products was not carried out	Elcheninov et al. 2017
Consortia with unidentified producers				A xanthan-degrading mixed bacterial culture from municipal wastewater	Extracellular xanthan-degrading activity	Experimental identification of products was not carried out	Obayashi and Gaudy (1973)
				Thermophilic, salt tolerant (3% NaC1, 45 °C) stable consortia NRRL B-14401 and NRRL B-18445 (from soil, USA)	Extracellular xanthan lyase and xanthan depolymerase. T_opt_ 50 °C, pH_opt_ 5.8. Xanthan-depolymerase is 100% stable for 20 min at 55 °C in the absence of salt, and at 60 °C in the presence of 3% NaCl. At 65 °C, 82% of activity was retained after 20 min. Inactivation at 70 °C	Pyruvoylated mannose, a branched tetrasaccharide with a terminal Δ4,5GlcA residue, and a branched non-pyruvoylated pentasaccharide	Cadmus et al. 1989

Isolation of bacterial xanthan-degrading strains is usually carried out by enrichment of environmental samples on a medium containing xanthan as the only carbon source. In these strains, the production of extracellular xanthan-specific enzymes is induced by xanthan. Most strains of bacteria that decompose xanthan have been isolated from samples of water, soil, sludge, or algobacterial mats (Table 2). However, some strains were found in human-made ecological niches such as *Paenibacillus taichungensis* I5 (VKM B-3510D) and *Cohnella* sp. 56 (VKM B-3672 D) obtained from spoiled xanthan thickener in the food industry (Denisenko et al. 2020, 2022) or *Bacillus pumilus* isolated from recovery water in the oil industry (Gimenez et al. 2018).

### Xanthan-utilizing microbial consortia

More commonly than pure bacterial cultures, consortia of xanthan-degrading microorganisms were obtained. Such consortia collectively produce the complex mixture of enzymes carrying out effective xanthan decomposition. In some cases, only one species in the consortium is responsible for xanthan degradation, while others stimulate the production of xanthan-degrading enzymes. For example, *Bacillus* sp. K11 exhibited higher activity in a mixed culture with soil microorganisms from various genera, particularly in the presence of strain K17, a *Flavobacterium* sp. (Cadmus et al. 1982); however, K11 was the sole producer of the xanthan-degrading enzymes. The different temperature optima found for xanthan-degrading activity, with or without 0.1 M NaCl (Table 2), indicated again the effect of xanthan conformation on enzymatic activity (Cadmus et al. 1982).

In the human gut microbiome, complete xanthan degradation was shown to be achieved through the synergistic activity of the *Ruminococcaceae* strain R.UCG13 and *Bacteroides intestinalis* (Ostrowski et al. 2022). Although *B. intestinalis* does not directly consume xanthan, it utilizes the metabolizable sugars in XTOS produced by depolymerizing enzymes of R.UCG13. The authors propose that the introduction of xanthan as a food supplement has contributed to the expansion of *Ruminococcaceae* and *B. intestinalis* in human gut consortia (Ostrowski et al. 2022) (Table 2).

### Degradation of xanthan by thermophilic and/or salt-tolerant microorganisms

Enzymes from xanthan-utilizing microorganisms isolated at high ionic strength are of great interest because they can decompose ordered xanthan at high salt content, for example, in brine. Strain *Microbacterium* sp. XT11, a mixed culture HD1 with a *Bacillus* sp. strain, and the strain of *Enterobacter* sp. nov. LB 37 were able to grow in media with 4–6% NaCl content (Qian et al. 2007; Hou et al. 1986; Chen et al. 2013). The aforementioned *Bacillus pumilus* was isolated from the extracted GEOR water with a high salt content.

Thermophilic xanthan-utilizing microorganisms are a natural source of thermostable xanthan-degrading enzymes, which can be used in technological processes occurring at elevated temperatures. The thermophilic aerobic strain *Brevibacillus thermoruber*, the facultatively anaerobic strain*s Anoxybacillus flavithermus*, *Anoxybacillus rupiensis*, and the anaerobic planctomycete *Thermogutta terrifontis* R1 were obtained from a microbial mat in hot springs (Derekova et al. 2007, 2008; Elcheninov et al. 2017) (Table 2). The thermophilic salt-tolerant consortia NRRL B-14401 and NRRL B-18445, producing extracellular xanthan-degrading enzymes active at 60–65 °C in brine, were isolated from soil by enrichment under high salt concentration (3% NaC1) and at elevated temperature (45 °C) (Cadmus et al. 1989).

All of the microorganisms and consortia described above, together with gene sequences in the databases homologous to already identified enzymes capable of decomposing or modifying xanthan, are potential candidates for obtaining enzymes for the development of new functional xanthan-based products for food, technical, and biomedical applications. However, due to the high specificity of carbohydrate-active enzymes (CAZymes) and the fact that even enzymes with closely similar primary structures can have strikingly different substrate or product specificities, it is necessary to characterize each suspected reading frame and the enzyme it codes for by wet lab work.

## Bacterial xanthan-specific enzymes

The xanthan-degrading enzyme systems of strains from *Bacillus*, *Paenibacillus*, *Microbacterium*, and *Ruminococcaceae* were investigated more closely. Generally, the bacterial systems for xanthan utilization are comprised of extracellular xanthan-specific lyase (EC 4.2.2.12) and xanthanase (endo-(1,4)-β-d-glucanase) (EC 3.2.1.-) for concerted depolymerization, and of additional intracellular accessory enzymes such as deacetylase and various exo-glycosidases that decompose XTOS into monomers. Here again, we observe a similarity to the degradation of lignocellulose, which requires the coordinated action of different specific CAZymes (Leis et al. 2018).

### Xanthan lyase

Xanthan lyase liberates the terminal mannose from the xanthan side chain through eliminative cleavage, generating Δ4,5-unsaturated glucuronic acid at the non-reducing end of the side chain. The presence of Δ4,5-unsaturated glucuronic acid in the products of xanthan degradation by extracellular xanthan-inducible *Bacillus* enzymes was first demonstrated by Lesley (1961). Later an extracellular xanthan lyase in addition to endo-β-(1,4)-d-glucanase was suggested (Sutherland 1982; Cadmus et al. 1989) and partially purified from *Corynebacterium*, *Bacillus*, and a mixed culture X23 (Sutherland 1987) (Table 2 and 3). Xanthan lyase was established as the starting enzyme in the pathway of xanthan degradation by *Bacillus* sp. GL1, demonstrating unsaturated saccharides as the first products formed during the decomposition of xanthan (Hashimoto et al. 1998).

**Table 3:** Characterized enzymes involved in xanthan degradation

Enzyme (protein database ID, CAZy family of a catalytic domain, EC number, PDB number)	Producer	MW	Substrate specificity	Maximal activity, stability	Reference
Xanthan lyase (no data)	A consortium of heat-stable, salt-tolerant bacteria	33 kDa	Specifically removed terminal pyruvoylated mannose residues but did not remove unsubstituted terminal mannose residues from xanthan side chains; did not hydrolyze *p*-nitrophenyl-β-d-mannose. Product: Δ4,5GlcA	pH 5.0, 60 °C in 0.05 M NaCI. Lost 50% activity at 60 °C without NaCl in < 1 h; retained 90% activity in 0.25 M NaCl after 1 h; Stable at 55 °C and unstable at 65 °C with or without NaCl	Ahlgren 1991
Xanthan lyase XL (AAG24953.1, PL8, EC 4.2.2.12)	Native producer *Paenibacillus alginolyticus* XL-1; heterologous host *E. coli*	100.8 kDa preproform with a signal peptide; 96.9 kDa mature form	The native and recombinant enzyme specifically removed pyruvoylated mannose from the native xanthan and showed no activity on depyruvoylated xanthan	The native enzyme was maximally active at pH 7.0, 55 °C, unstable above 55 °C; the recombinant enzyme was maximally active at pH 7.0, 65 °C, unstable above 65 °C	Ruijssenaars et al. 1999, 2000
Xanthan lyase BXL (BAB21059.1, PL8, EC 4.2.2.12, PDB 1J0M, 1X1H, 2E22)	Native producer *Bacillus* sp. GL1; heterologous host *E. coli*	99 kDa preproform with a signal peptide; 97 kDa precursor; 75 kDa mature form	The native and recombinant 75 kDa enzymes specifically removed pyruvoylated mannose from the native xanthan; 69% activity was retained on partially depyruvoylated xanthan. Acetylation has no effect. No activity on hyaluronate, chondroitin, alginate, fucoidan, gellan, and pectin	pH 5.5, 50 °C. Stable at pH 6.5–9.0, 4–37 °C; 60% activity was lost after 10 min at 45 °C, pH 7.0. Slightly affected by metal ions. Inhibited by 150 mM NaCl and KCl	Hashimoto et al. 1998, 2001, Maruyama et al. 2007
Xanthan lyase MXL (ALX66168.1, PL8, EC 4.2.2.12). *Mi*XBM (PDB 7EHG)	*Microbacterium* sp. XT11	110 kDa	Specifically removed pyruvoylated mannose from the native xanthan; 50% activity remained on partially depyruvated xanthan; acetylation degree has no effect. The activity on XTOS has not been studied	pH 6.0–6.5, 40 °C. Stable at 20–40 °C, pH 5.5–11.0; no activity at 50 °C. 90% of activity remained after 2 h at pH 11.0. Enhanced by 10 mM K^+^, Ca^2+^, Na^+^, Mg^2+^, Mn^2+^; Li^+^, inhibited by Zn^2+^, Cu^2+^	Yang et al. 2014
Xanthan lyase PXL (AXR85425.1, PL8, EC 4.2.2.12), PDB 6F2P	*Paenibacillus nanensis*	113 kDa	Specific for both unmodified mannose and pyruvoylated mannose of xanthan	Maximally active at pH 7.6, 40 °C. Destabilized upon Ca^2+^ removal	Jensen et al. 2019
Xanthan lyase PL8 (European Nucleotide Archive, BioProject PRJEB44146, EC 4.2.2.12)	Native producer R.UCG13 strain of *Ruminococcaceae*; heterologous host *E. coli*	90 kDa	Showed no activity on native xanthan but was active on XTOS produced by RuGH5a	No data	Ostrowski et al. 2022
Xanthan-specific endo-(1,4)-β-D-glucanase (no data)	Bacterial consortium (NRRL B-14401)	170 kDa	Cleaved the endo-β-(1,4)-glycosidic linkages of the native xanthan in disordered conformation and CMC	pH 6.0, 45 °C. Stable after 6 h at 50 °C with CMC; rapidly inactivated without CMC. A purified enzyme was less thermostable than a crude enzyme	Ahlgren 1993
Xanthan-specific β-glucanase (no data)	*Bacillus* strain R	No data	Activity (%) on native xanthan (100), CMC (96), β-(1,3)-linked glucan (36); activity on deacetylated and depyruvoylated xanthan	No data	Sutherland 1982
Xanthan-specific endo-(1,4)-β-d-glucanase (no data, EC 3.2.1.-)	*Bacillus* sp. GL1	173 kDa	Cleaved endo-β-(1,4)-glycosidic linkages of the lyase-treated xanthan backbone at the reducing end of the branching glucose; 8.2% activity on native xanthan; 1.6% activity on CMC	pH 6.0, 45 °C. Stable below 40 °C. 30% of activity was lost after incubation at 40 °C, pH 6.0 for 10 min	Nankai et al. 1999
Xanthan-specific endo-processive (1,4)-β-d-glucanase *Psp*Xan*9* (AXR85426.1, GH9, EC 3.2.1.-, PDB 6FHN)	Native producer *Paenibacillus* sp. 62,047; heterologous host* B. subtilis*	110 kDa	Cleaved endo-β-(1,4)-glycosidic linkages of the lyase-treated xanthan backbone at the reducing end of the branching glucose; the activity was highly dependent on Ca^2+^	pH 7.0–7.5, 63 °C, 2 mM CaCl_2_. Stable after 1 h at 60 °C in the presence of Ca^2+^; 75% activity retained after 48 h at 54 °C. Absence of Ca^2+^ 40% of activity was lost after 1 h at 45 °C	Moroz et al. 2018
Endo-glucanase with activity on xanthan (ASV74163.1, DUF1080, EC 3.2.1.-)	Native producer *Thermogutta. terrifontis* R1(T); heterologous host *Penicillium verruculosum* 537 (ΔniaD)	23.7 kDa	Activity (U/mg) on native xanthan (0.12), CMC (0.58), β-glucan (0.56), curdlan (0.12), lichenan (1.48), laminarin (0.17), galactomannan (0.12), xyloglucan (0.18). Xanthan lyase increased the efficiency of xanthan hydrolysis	pH 4.0, 55 °C; 90% of activity retained at 50–60 °C, pH 3–5; 30% of activity retained at pH 2 and 6, and at 80 °C	Denisenko et al. 2021
Xanthan-specific endo-(1,4)-β-d-glucanase *Mi*GH (WP_067195711, GH5, EC 3.2.1.-)	*Microbacterium* sp. XT11	47 kDa	Cleaved endo-β-(1,4)-glycosidic linkages of the native xanthan was not active on the lyase-treated xanthan	pH 6.0, 35–40 °C. Unstable at 40 °C	Li et al. 2009
Xanthan-specific endo-(1,4)-β-d-glucanase *Mi*Xen (ALX66163.1, GH9, EC 3.2.1.-)	Native producer *Microbacterium* sp. XT11; heterologous host *E. coli*	101 kDa	Cleaved endo-β-(1,4)-glucosidic linkages of the highly ordered native, pyruvate-free, acetyl-free, acetyl- and pyruvate-free, terminal mannosyl residue-free xanthans, and CMC	pH 7.5–8.0, 40–45 °C on CMC. > 70% of activity retained after 30 min at 20–45 °C	Yang et al. 2019
Endo-xanthanase *Ru*GH5a (European Nucleotide Archive, BioProject PRJEB44146, GH5, EC 3.2.1.-)	Native producer R.UCG13 strain of *Ruminococcaceae*; heterologous host *E. coli*	230 kDa recombinant form	Cleaved endo-β-(1,4)-glycosidic linkage of the xanthan with various acetylation (including di-acetylation) and pyruvoylation at the reducing end of the non-branching glucose; comparable specificity to native and lyase-treated xanthan	Maximally active at pH 6.0, 35–40 °C	Ostrowski et al. 2022
Acetyl xylan esterase AXE3 (ADZ98864.1, CE1, EC 3.1.1.72)	*Myceliophthora thermophila* C1	31.5 kDa	Removed the acetyl groups from the inner mannose but not from the outer mannose residues. Active only on disordered xanthan	53 °C in salt-free conditions. At optimal conditions, 70% of all acetyl groups on the inner mannose units were hydrolyzed	Kool 2014
Acetyl pectin esterase YesY (CAB12526.1, CE12, 3.1.1.-)	*Bacillus subtilis* 168	24.6 kDa, no signal sequence	Specifically removed the acetyl groups at the outer mannose residues of xanthan; the activity was not influenced by the xanthan conformation	pH 6.3, 55 °C	Kool 2014
Unsaturated glucuronyl hydrolase (BAA84216.1, GH88, 3.2.1.179)	*Bacillus* sp. GL1	42.9 kDa	Cleaved xanthan trisaccharide (Δ4,5GlcA-β-(1,2)-d-Man-α-(1,3)-d-Glc). Products: d-Man-α-(1,3)-d-Glc; Δ4,5GlcA	pH 6.0–6.5, 55 °C	Hashimoto et al. 1999

Ahlgren (1991) reported on xanthan lyase releasing only pyruvoylated mannose from the side chains (Table 3). Xanthan lyases from various bacteria were then characterized and classified as members of polysaccharide lyase family 8 (PL8) in the CAZy database (Hashimoto et al. 1998, 2001; Yang et al. 2014; Ruijssenaars et al. 1999, 2000; Jensen et al. 2019; Drula et al. 2022) (Table 3). Except for PXL from *P. nanensis*, also able to recognize non-pyruvoylated mannose, all characterized enzymes exhibit specificity toward pyruvoylated mannose. The resolved spatial structures of BXL from the *Bacillus* sp. GL1 strain and PXL from *P. nanensis* are similar to that of hyaluronate and chondroitin lyases from the PL8 family. The catalytic mechanism is common among all PL8 enzymes, while substrate recognition varies (Hashimoto et al. 1998, 2001; Maruyama et al. 2007). Structural studies of PXL have identified determinants that contribute to its promiscuous activity toward both pyruvoylated and non-pyruvoylated mannose (Jensen et al. 2019).

An interesting observation is that the *Klebsiella* K5 phage produces an enzyme that can cleave a 4,6-Pyr-β-d-Man-(1 → 4)-β-d-GlcA linkage present in the backbone of the extremely viscous capsular polysaccharide of this bacterium. The enzyme shows limited activity on xanthan which has a similar linkage in the side chain (Sutherland 1999). Although the putative lyase has not been identified, it was shown that one of the tail fiber proteins of *Klebsiella* K5-2 phage has a high level of sequence similarity with bacterial polysaccharide lyases.

Thus, bacterial xanthan lyase performs a crucial first step in the decomposition of xanthan, shortening its side chains by removing the terminal mannosyl residues. This reduces the viscosity of the xanthan solution by about 60% (Table 1, 3) (Ahlgren 1991). It is reasonable to assume that the removal of the terminal pyruvoylated mannosyl residues destabilizes the double-stranded helix conformation of the native xanthan by weakening the inter-strand association. Since xanthan fibers are assembled from smaller subunits (Holzwarth and Prestridge 1977) (Fig. 2A), xanthan lyase treatment leads to fiber fragmentation and thus to a decrease in viscosity. In addition, xanthan subunits become more accessible for further degradation by xanthanases. Consequently, xanthan lyase prepares the substrate, facilitating xanthanase access to the xanthan backbone.

### Xanthanase

Bacterial xanthanases cleave the β-(1,4)-glucosidic linkages in the xanthan backbone in an endo-mode (Table 1 and 3). The rate of hydrolysis of non-modified xanthan by xanthanases is affected by ionic strength in the environment. The purified bacterial xanthanase shows maximal activity in a salt-free solution. The addition of NaCl or KCl is associated with the transition of xanthan from a disordered to an ordered conformation and consequently affects the rate of hydrolysis negatively (Ahlgren 1993). Since the highly ordered conformation of native xanthan shields its backbone from depolymerization, most bacterial xanthanases exhibit little or no activity against native xanthan but hydrolyze with high specific activity lyase-treated xanthan (Cadmus et al. 1989; Nankai et al. 1999; Moroz et al. 2018) (Table 3). Nevertheless, some xanthanases do not require pretreatment with xanthan lyases. For example, endo-xanthanase *Mi*Xen from *Microbacterium* sp. XT11 exhibited high specific activity and strong affinity for highly ordered xanthan, although it also showed some activity against xanthan lacking terminal mannosyl residues (Yang et al. 2019) (Table 3). It is noteworthy that *Microbacterium* sp. XT11 is tolerant to 4% NaCl—under these conditions xanthan exists in an ordered conformation. *Mi*Xen, although a GH9 enzyme, has less than 25% sequence similarity to other characterized GH9 enzymes and may belong to a novel branch within the GH9 enzyme family (Yang et al. 2019). Another endo-xanthanase from *Microbacterium* sp. XT11, the GH5 family enzyme *Mi*GH, specifically cleaves the backbone of native xanthan but does not exhibit any activity on the polytetramer produced by xanthan lyase (Li et al. 2009).

Glycoside hydrolase RuGH5a, a GH5 family enzyme derived from the R.UCG13 strain of *Ruminococcaceae*, exhibits activity on both native and lyase-treated xanthan (Ostrowski et al. 2022). It hydrolyzed the xanthan backbone at the reducing end of the undecorated glucose residue (Ostrowski et al. 2022). This is in contrast to GH9 xanthanase *Psp*Xan9 from *Paenibacillus* sp. 62,047 (closely related to *P. nanensis*) (Moroz et al. 2018) or the xanthanase from *Bacillus* sp.GL1 (Nankai et al. 1999) which cleave the β-(1,4)-linkages of the xanthan backbone at the reducing end of the sidechain-carrying glucose residue, thus demonstrating a different reaction mechanism.

The endo-xanthanase from the thermophilic planctomycete *T. terrifontis* R1 is particularly interesting due to its classification within the family DUF1080, which displays structural resemblance to GH16 endo-(1,3–1,4)-β-d-glucanases, except for the structural details of the catalytic cleft. Xanthanase DUF1080 exhibited activity on native xanthan; however, removal of the terminal mannosyl residue by xanthan lyase enhanced the hydrolytic efficiency of DUF1080. Furthermore, apart from xanthan, this enzyme hydrolyzed CMC, β-glucan, curdlan, lichenan, laminarin, galactomannan, and xyloglucan. The advantageous combination of thermostability at 60 °C, broad substrate specificity, and small size (23.7 kDa) positions DUF1080 as a promising candidate for various technical applications (Denisenko et al. 2021).

It should be noted that all so far known bacterial xanthanases belong to the CAZy families GH9 and GH5 and the DUF1080 family (similar to GH16). These families also comprise xyloglucan-specific endo-(1,4)-β-d-glucanases. Xyloglucan is a soluble and highly viscous branched polysaccharide that, like xanthan, has a β-(1,4)-d-glucan backbone chain. However, due to uncharged side chains xyloglucan is more vulnerable to enzymatic digestion (Zavyalov et al. 2019; Rykov et al. 2019).

### Xanthan-binding modules

An essential characteristic of bacterial xanthan lyases and xanthanases is the presence of xanthan-binding modules (XBMs). For example, the R.UCG13 xanthanase RuGH5a has three uncharacterized domains (UDs A-C) annotated as sugar-binding lectins. In addition, UD-A and UD-C have been predicted as carbohydrate binding modules (CBM) belonging to family 11. All three UDs play an important role in maintaining enzyme stability and catalytic activity and are assumed to be XBMs (Ostrowski et al. 2022).

XBMs belonging to the CBM family 84 are found in xanthan lyase PXL and xanthanase *Psp*Xan9 from *Paenibacillus* sp. 62,047, as well as xanthan lyase MXL and endo-xanthanase *Mi*Xen from *Microbacterium* sp. XT11 (Yang et al. 2014; Jensen et al. 2019; Moroz et al. 2018). Notably, PXL harbors an additional terminal xanthan-binding module that remains unclassified.

Deletion of the XBMs from PXL and *Mi*Xen resulted in a significant reduction in the activity on xanthan and an elevation in K_m_ values of the mutants compared to native enzymes (Jensen et al. 2019; Yang et al. 2019). However, the removal of XBM from *Psp*Xan9 did not lead to a decrease in the activity of this xanthanase in the presence of xanthan lyase (Moroz et al. 2018).

A study of the properties of the *Mi*Xen catalytic module, either fused with the XBM originating from *Psp*Xan9, or with its own XBM has demonstrated that both XBMs increase the catalytic activity of xanthanase by enhancing the enzyme–substrate interaction. However, *Mi*Xen fused with *Psp*XBM showed a higher affinity for the substrate than *Mi*Xen equipped with its native XBM. Furthermore, the hybrid xanthanase has increased thermostability and greater affinity for the substrate, leading to enhanced enzymatic efficiency (Ni et al. 2023). The use of XBMs for engineering xanthanases with altered or improved characteristics may find applications in the development of technologies for producing functional XTOS, such as oligosaccharides with antioxidant activity, as proposed by Ni et al. (2023).

Thus, xanthan lyase and endo-xanthanase serve as key enzymes in the bacterial xanthan-degrading systems. The presence of XBMs in these enzymes maintains their proximity to potential recognition sites on the xanthan molecule, thereby increasing enzymatic activity toward the substrate. However, the complete degradation of xanthan into sugars necessitates the involvement of accessory enzymes targeting the linkages within the XTOS generated through the activities of the xanthan lyase and xanthanase (Table 4). Evidently, different bacterial species possess distinct sets of enzymes capable of decomposing xanthan.

**Table 4:** Microbial enzyme systems for xanthan degradation and utilization

Microorganisms	Enzymes participating in the complete degradation of xanthan	References
*Bacillus* sp. GL1	Extracellular: xanthan lyase, xanthan-specific endo-(1,4)-β-glucanase Intracellular: β-glucosidase, unsaturated glucuronyl hydrolase, deacetylase, α-mannosidase	Hashimoto et al. 1998, 1999, Nankai et al. 1999, 2002
*Microbacterium* sp. XT11	Extracellular: PL8 polysaccharide lyase (prot. ID LX1-1GL001102), GH9 xanthan-specific endo-(1,4)-β-glucanase *Mi*Xen (prot. ID LX1-1GL001095), xanthan-specific endo-(1,4)-β-glucanase *Mi*GH Intracellular: GH3 β-glucosidase (prot. ID LX1-1GL001100), α-mannosidase GH38 (prot. ID LX1-1GL001101) ATP-binding cassette (ABC)-type sugar transport complex (prot. ID LX1-1GL001097, LX1-1GL001098, LX1-1GL001099)	Li et al. 2009, Yang et al. 2016, 2019, Sun et al. 2019, Gu et al. 2022
*Thermogutta terrifontis* R1	Extracellular: two GH5 endomannanases/β-mannosidases (THTE_3333 and THTE_3787), GH2 β-glucuronidase THTE_2104, GH38 α-mannosidase (THTE_2605) or GH36 α-galactosidase (THTE_1560), DUF1080 endo-xanthanase (THTE_1561), glycosidases GH2 or GH5 (THTE_1171)	Denisenko et al. 2021, Elcheninov et al. 2017
*Ruminococcaceae* strain R.UCG13	GH5 endo-xanthanase *Ru*GH5a, CE-A and CE-B carbohydrate esterases, PL8 XTOS-specific xanthan lyase, GH88 unsaturated glucuronyl hydrolase, GH38-A and GH38-B α-mannosidases, GH94 cellobiose phosphorylase	Ostrowski et al. 2022
*B. intestinalis*	R.UCG13 XTOS-degrading enzymes: CE domain of PL-CE, PL unknown polysaccharide lyase, GH88 unsaturated glucuronyl hydrolase, GH92 α-mannosidase, GH3 β-glucosidase	Ostrowski et al. 2022

## Bacterial enzymatic pathways for xanthan degradation

**Fig. 3:**
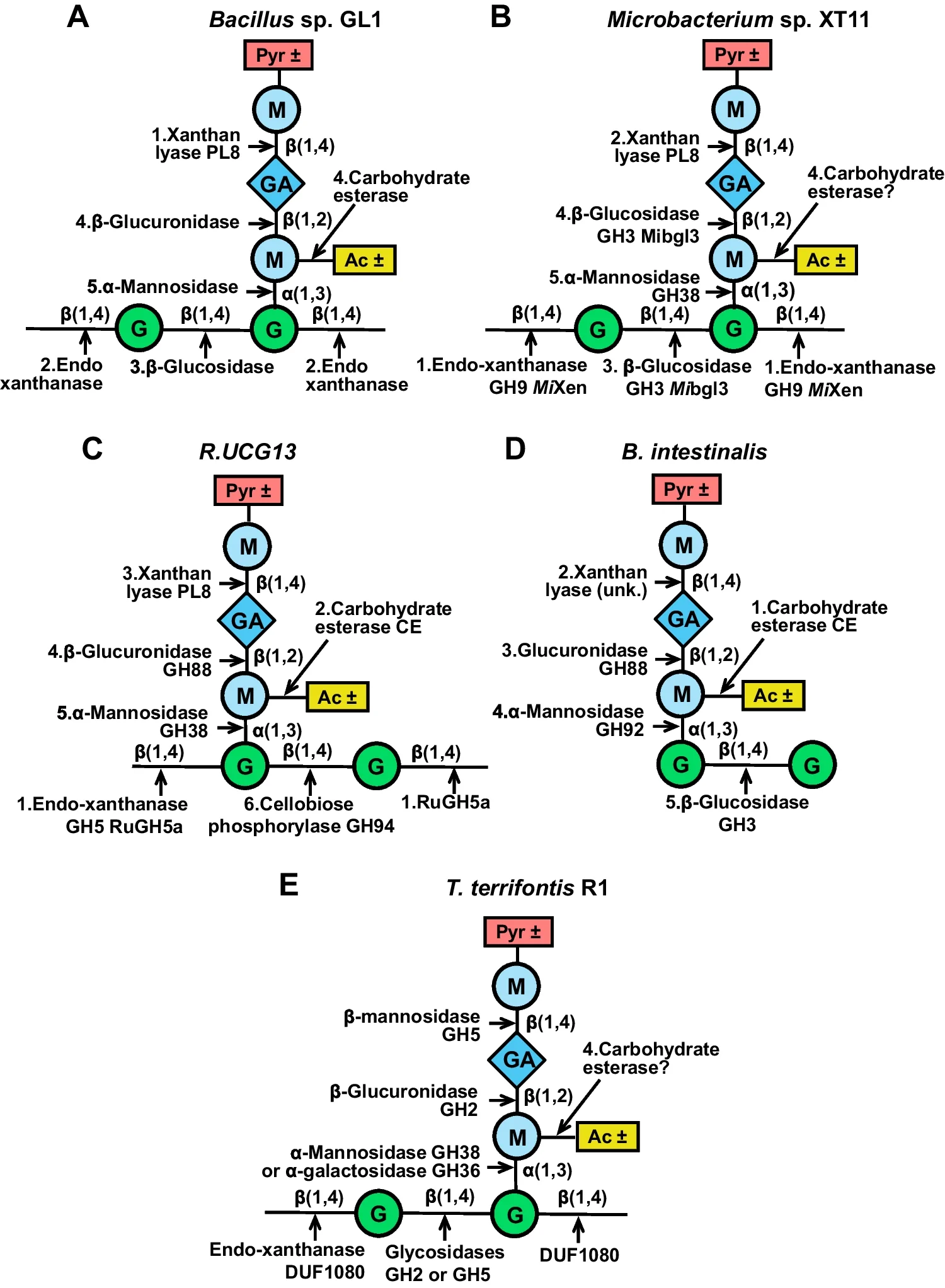
Enzymatic pathways for xanthan degradation in *Bacillus* sp. GL1 (**A**), *Microbacterium* sp. XT11 (**B**), R.UCG13 (**C**), *B. intestinalis* (**D**), *T. terrifontis* R1 (**E**). The cleavage sites of xanthan-degrading enzymes are indicated by arrows. The numbers show the succession of the reactions, where these are known. G, glucose; GA, glucuronic acid; M, mannose; Pyr, pyruvoylation; Ac, acetylation; ± , variable

The first complete xanthan decomposition pathway was described for *Bacillus* sp. strain GL1 through the analysis of its xanthan degradation products (Nankai et al. 1999) (Fig. 3A). At the initial stage, extracellular xanthan lyase (Hashimoto et al. 1998) liberates pyruvoylated terminal mannose residues from the side chains of xanthan. Subsequently, extracellular xanthan-specific β-d-glucanase hydrolyzes the polymer to tetramers. The resultant tetramers are proposed to enter the cell, where they are subject to consecutive cleavage by β-d-glucosidase, unsaturated glucuronyl hydrolase (Hashimoto et al. 1999), and α-d-mannosidase (Nankai et al. 2002) for complete monomerization. Acetyl groups are likely removed by carbohydrate esterase during the conversion of trimer-units (Δ4,5-GlcA–ManAc–Glc) to dimer-units (Man–Glc) (Nankai et al. 1999).

A similar pathway of xanthan degradation was proposed for *Microbacterium* sp. XT11 through genome (Yang et al. 2016) and proteome analysis (Sun et al. 2019). Genes encoding key enzymes involved in this pathway, namely GH9 endo-xanthanase *Mi*Xen, PL8 polysaccharide lyase MXL, GH38 α-mannosidase, GH3 β-glucosidase *Mi*bgl3 (Gu et al. 2022), and ATP-binding cassette (ABC) transporters, are organized in a gene cluster (Yang et al. 2016). Unlike extracellular endo-xanthanase *Mi*Xen and xanthan lyase MXL, which are equipped with signal peptides and XBMs, the GH3 and GH38 XTOS-degrading enzymes have neither signal peptides nor XBMs, indicating their intracellular location (Yang et al. 2016). The expression of the cluster of genes related to xanthan breakdown and the synthesis of the corresponding proteins by *Microbacterium* sp. XT11 grown on xanthan were confirmed by qRT-PCR and appropriate enzyme assays, respectively, which is strong evidence for the involvement of the identified genes in xanthan degradation (Sun et al. 2019).

As mentioned in the previous chapter, *Mi*Xen, the major endo-xanthanase of *Microbacterium* sp. XT11, was able to efficiently hydrolyze highly ordered xanthan (Yang et al. 2019). Another xanthanase *Mi*GH was not active on lyase-treated xanthan (Li et al. 2009) (Table 3). This suggests that in this case release of the terminal mannose residue by the lyase may not be the initial step in xanthan degradation, as endo-xanthanases potentially act first. After extracellular degradation of xanthan, the resulting XTOS tetramers are taken up into the cells by ABC transporters. They are subsequently hydrolyzed by the intracellular enzymes β-d-glucosidase *Mi*bgl3 and α-d-mannosidase (Yang et al. 2016) (Fig. 3B).

The complete saccharification of xanthan in *Bacillus* and *Microbacterium* involves the common reactions catalyzed by extracellular xanthan-specific lyase and β-d-glucanase, as well as intracellular β-d-glucosidase and α-d-mannosidase. The release of unsaturated glucuronic acid is catalyzed by glucuronidase in *Bacillus* or by β-d-glucosidase in *Microbacterium* (Table 4, Fig. 3). The study of the xanthan degradation pathway in *Microbacterium* sp. XT11 revealed the important role of transporters in this process.

Another pathway for xanthan degradation was predicted by analyzing the transcriptomes of *T. terrifontis* R1 grown on xanthan and by identifying the genes activated in the presence of this polysaccharide. Among these genes were *thte_1561*, which encodes the endo-xanthanase DUF1080 mentioned earlier, *thte_2104* encoding a putative extracellular GH2 β-glucuronidase, *thte_2605*, and *thte_1560*, which encode a GH38 α-mannosidase and a GH36 α-galactosidase, respectively. Notably, no homologs of PL8 xanthan lyase were found through in silico analysis, suggesting the possible substitution of this enzyme with an extracellular GH5 β-mannosidase that releases terminal β-d-mannose from the xanthan side chains. The upregulation of *thte_3333* and *thte_3787* genes encoding GH5 β-mannosidase in the xanthan cultures further supports their involvement in xanthan decomposition (Table 4). However, it should be noted that these enzymes predicted by in silico analysis have not yet been characterized biochemically (except endo-xanthanase DUF1080) (Elcheninov et al. 2017; Denisenko et al. 2021) (Fig. 3E).

Metagenomic analysis of the gut microbiomes of modern humans revealed clusters of genes responsible for the degradation of xanthan associated with two classes of bacteria—*Clostridia* (specifically the R.UCG13 strain of *Ruminococcaceae*) and *Bacteroidia* (*B. intestinalis*). A locus related to R.UCG13 was expressed during growth on xanthan and included genes encoding a PL8 polysaccharide lyase, two putative GH5 xanthanases, a GH88 unsaturated glucuronyl hydrolase, a GH94 cellobiose phosphorylase, two GH38 α-mannosidases, and two CE carbohydrate esterases. Additionally, the locus contained four putative genes associated with ABC transporters and a gene encoding a CBM of family 11 (Ostrowski et al. 2022).

In contrast to known xanthan lyases (Table 3), PL8 from R.UCG13 showed no activity against native xanthan but released terminal mannose from side chains of XTOS pentamers, the product of xanthanase RuGH5a activity. This again emphasizes the importance of wet-lab investigations of predicted open reading frames. Both identified CEs were found to deacetylate XTOS. The resulting tetramer was subsequently hydrolyzed by the GH88 and GH38 enzymes, leading to the formation of cellobiose. Finally, the degradation of xanthan was completed by the action of a GH94 cellobiose phosphorylase (Ostrowski et al. 2022) (Fig. 3C).

The genes present in the polysaccharide utilization locus (PUL) of *B. intestinalis* presumably encode a polysaccharide lyase-carbohydrate esterase (PL-CE), glycoside hydrolases GH3, GH5, GH88, GH92, and the TonB-dependent transporters of sugars. Expression of the PUL genes was induced by the cultivation of *B. intestinalis* on XTOS (Ostrowski et al 2022; Pollet et al. 2021). The CE module of the PL-CE enzyme was found to deacetylate XTOS, while no lyase activity was detected with xanthan or XTOS as substrates. Nevertheless, the *B. intestinalis* cell lysate demonstrated lyase activity against XTOS, but the enzyme responsible for this activity was not identified.

Intracellular *B. intestinalis* GH88, GH92, and GH3 enzymes sequentially degraded XTOS tetramers produced by xanthanase *Ru*GH5a and a PL8 xanthan lyase. These results suggest that in the human gut, the R.UCG13 enzymes hydrolyze xanthan into the tetramers, which are further metabolized by R.UCG13 or *B. intestinalis* (Table 4, Fig. 3D) (Ostrowski et al. 2022). Notably, *B. intestinalis* did not utilize tetramers generated by the GH9 endo-xanthanase from *P. nanensis* and PL8. Altogether, this indicated that the transport system and/or XTOS-degrading enzymes of *B. intestinalis* are highly specific for the XTOS produced with *Ru*GH5a, involving interspecies synergism (Ostrowski et al. 2022; Martens et al. 2021).

The gene cluster responsible for xanthan degradation in gut strain R.UCG13 was proposed to have initially evolved to break down a diverse array of substrates, including the EPS produced by endogenous gut microorganisms. Subsequently, with the widespread incorporation of xanthan into various food products, this gene cluster underwent adaptive evolution to accommodate the degradation of this novel dietary supplement (Ostrowski et al. 2022).

**Fig. 4:**
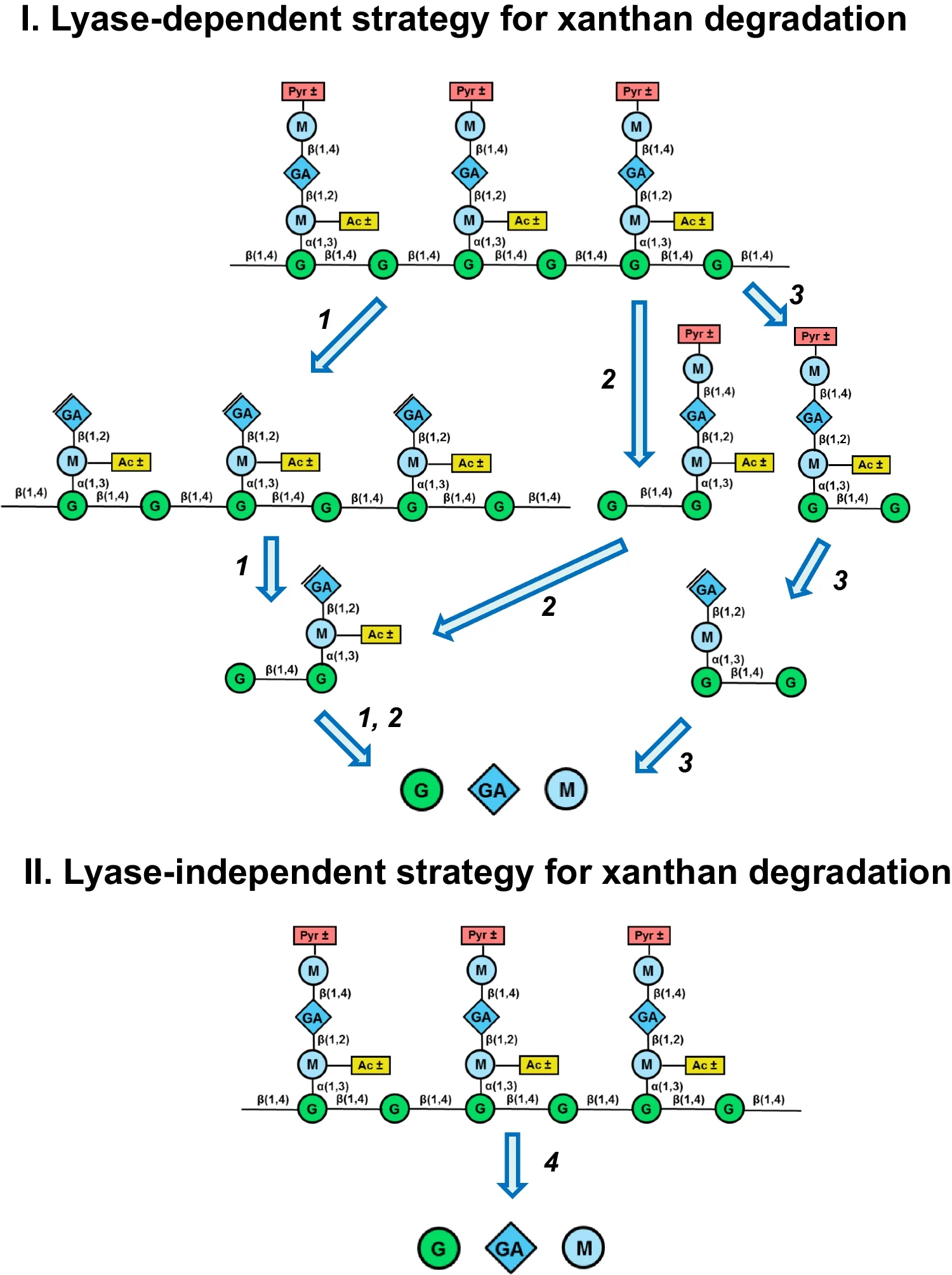
Two different strategies employed by bacteria for xanthan degradation. *Bacillus* sp. GL1 (1), *Microbacterium* sp. XT11 (2), R.UCG13 (3), *T. terrifontis* R1 (4). G, glucose; GA, glucuronic acid; M, mannose; Pyr, pyruvoylation; Ac, acetylation; ± , variable

These findings reveal two basic strategies employed by bacteria for xanthan degradation (Fig. 4). The first strategy observed in members of *Bacilli*, *Actinomycetia*, and *Clostridia* entails the enzymatic depolymerization of xanthan via the highly specialized extracellular enzymes xanthan lyase (PL8) and xanthan-specific endo-(1,4)-β-d-glucanase (GH9 or GH5). This initial degradation is followed by intracellular saccharification carried out by glycosidases. In addition, carbohydrate esterases are involved in the removal of acetyl groups from xanthan side chains. Typically, the genes responsible for xanthan degradation are organized in clusters. Variations within this strategy arise depending on what happens first, depyruvoylation (in *Bacilli*), or xanthan backbone splitting (in R.UCG13 and most probably in *Microbacterium*).

The second strategy predicted after in silico analysis of the *Thermogutta* transcriptome sequence presumably involves non-specific enzymes for xanthan degradation, such as GH5 β-mannosidases and a promiscuous xanthanase DUF1080 (Elcheninov et al. 2017; Denisenko et al. 2021). This bacterium from the class *Planctomycetia* phylogenetically differs from other bacteria that decompose xanthan. It has to be noted that most of the *Thermogutta* xanthan-degrading enzymes have not yet been biochemically characterized.

## Conclusion

Although xanthan is a well-characterized complex polysaccharide, recent studies have revealed important new findings concerning the function of the xanthan-degrading enzymes and their interaction with the substrate. The key problem of xanthan biodegradation is that the double-stranded helix conformation of native xanthan hinders enzyme access to the polysaccharide’s backbone chain. Changes in conformation, influenced by side chain structures and environmental conditions, can affect the activity of enzymes that decompose xanthan. Therefore, certain bacteria employ specialized enzyme systems for xanthan degradation, where initial degradation steps reduce substrate viscosity, thus facilitating further saccharification.

Analysis of the xanthan degradation pathways and screening of bacterial and metagenomes allowed us to predict at least two basically distinct strategies used by bacteria for xanthan degradation. One relies on lyases and is found in *Bacilli*, *Clostridia*, and *Actinomycetia*. It is characterized by a clustered organization of the genes responsible for xanthan degradation. The other is a lyase-independent enzyme system identified in *Planctomycetia*. The absence of a uniform set of homologous xanthan-degrading enzymes and of a uniform breakdown pathway suggests the independent evolution of enzyme systems for xanthan degradation across various classes of bacteria. Notably, the discovery of xanthan-degrading bacteria in the human gut microbiota indicates the evolutionary recent adaptation of the intestinal microflora to new food additives.

Thus, recent insights into the microbial enzyme systems of xanthan degradation enhance our understanding of xanthan decomposition processes in microorganisms. Using this knowledge on the function of single enzymes paves the way for xanthan modification and hence for novel applications in the food, biomedical, and technical fields. Enzymes like endo-xanthanase, lyase, glucuronidase, or esterase hold the potential for tailoring xanthan’s rheological properties to specific biotechnological requirements, reducing viscosity after technical use, or efficiently producing XTOS for beneficial food or feed supplements. Enzymatically modified xanthan derivatives could find application in various industrial sectors, including novel functional composites. The high industrial potential of some xanthan-degrading enzymes underscores the need to develop cost-effective and environmentally friendly microbial systems for the large-scale production of these enzymes.
